# Supplementary material for: Effects of Energy Drink Acute Assumption in Gastrointestinal Tract of Rats
Source: Nutrients. 2022 May 4;14(9):1928. doi: 10.3390/nu14091928 (PMC9105126; doi:10.3390/nu14091928)
Supplement: Supplementary file 1 [file nutrients-14-01928-s001.zip › nutrients-1665446-supplementary.pdf]

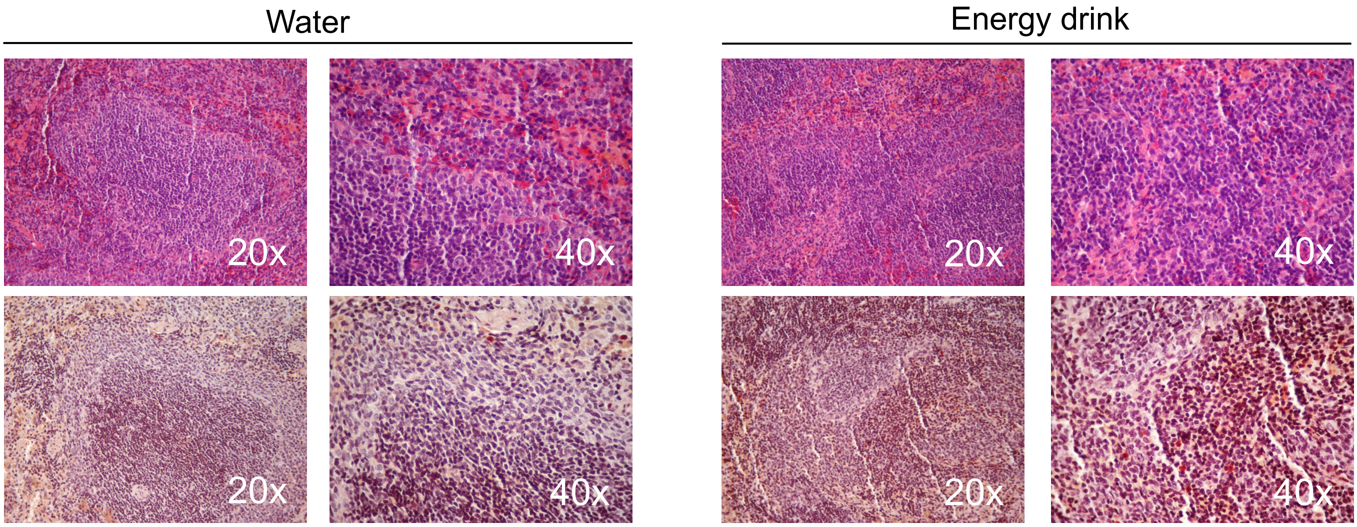

Figure S1. Caffeine triggers eosinophilic infiltration in the spleen in rats. Representative histological staining of spleen tissue of rats after 5 days of administration of water or energy drink Upper panels: H&M staining. Lower panels: Pagoda red staining.

Figure S1
